# Characterization of slow cycling corneal limbal epithelial cells identifies putative stem cell markers

**DOI:** 10.1038/s41598-017-04006-y

**Published:** 2017-06-19

**Authors:** R. Sartaj, C. Zhang, P. Wan, Z. Pasha, V. Guaiquil, A. Liu, J. Liu, Y. Luo, E. Fuchs, M. I. Rosenblatt

**Affiliations:** 1 University of Illinois, Chicago, USA; 2 Weill Cornell Medical College, New York, USA; 3 The Rockefeller University, New York, USA

## Abstract

In order to identify reliable markers of corneal epithelial stem cells, we employed an inducible transgenic “pulse-chase” murine model (K5Tta × TRE-H2BGFP) to localize, purify, and characterize slow cycling cells in the cornea. The retention of GFP labeling in slowly dividing cells allowed for localization of these cells to the corneal limbus and their subsequent purification by FACS. Transcriptome analysis from slow cycling cells identified differentially expressed genes when comparing to GFP^-^ faster-dividing cells. RNA-Seq data from corneal epithelium were compared to epidermal hair follicle stem cell RNA-Seq to identify genes representing common putative stem cell markers or determinants, which included Sox9, Fzd7, Actn1, Anxa3 and Krt17. Overlapping retention of GFP and immunohistochemical expression of Krt15, ΔNp63, Sox9, Actn1, Fzd7 and Krt17 were observed in our transgenic model. Our analysis presents an array of novel genes as putative corneal stem cell markers.

## Introduction

Loss of the regenerative capacity of the ocular surface through the absence of corneal epithelial stem cells is a potentially blinding condition. Lack of definitive molecular markers to reproducibility locate, purify and expand corneal epithelial stem cells has hampered the ability to understand their biology and to use these cells for therapeutic transplantation.

Stem cells from the cornea reside between the corneal periphery and the conjunctiva, known as the limbus. Limbal stem cells (LSCs) are clonogenic, regenerating new tissue *in vivo* and *in vitro*^1^ exhibiting slow cycling phenotypic characteristics^2^. Classically, these cells have been characterized by the ability to retain tritiated thymidine or bromodeoxyurdine (BrdU) for long periods, yet have high proliferative potential^3–7^. More recently, a transgenic system that can genetically label slow cycling cells with GFP has been used to identify label retaining cells (LRCs) in skin^8,9^, sweat glands^10^, salivary glands^11^ and the cornea^12^. In this model, one parental strain harbors the H2B-GFP transgene under the control of a tetracycline (doxycycline; dox) regulatory element (TRE), creating a tet-off system. The second strain expresses a transcription factor regulated by tetracycline (tTA) under the control of a cell-type-specific Keratin 5 (K5) promoter. Thus, K5 expressing cells will have nuclear GFP labeling in the “pulse” period, which is then turned off with dox administration, beginning the “chase” period.

This approach allowed for the purification of cells and their subsequent molecular characterization of LRCs using next generation sequencing (NGS), yielding genes marking (and in some cases determining) “stemness”. Thus, the molecular markers are identified without a pre-conceived notion as to which genes may be related to stemness. Comparison of these data from corneal epithelium with prior similar characterization of hair follicle stem cells (HFSCs)^13^ were performed to identify common stem cell markers in these developmentally related tissues.

## Methods

### Murine Models

All animal experiments were approved by the Institutional Animal Care and Use Committee (IACUC) from Weill Cornell Medical College, in accordance with the US National Institutes of Heath Guide for the Care and Use of Laboratory Animals and guidelines of the Association for Research in Vision and Ophthalmology Statement for the use of Animals in Ophthalmic and Vision Research. Wildtype (WT) CD1 mice were obtained from Jackson Laboratories (Bar Harbor, ME). The double transgenic mice were obtained by crossing heterozygous K5Tta (FVB) × TRE-H2BGFP (CD1) from E. Fuchs laboratory at the Rockefeller University^8^.

### Wholemount Corneal Imaging and K5Tta × TRE-H2BGFP Slit lamp Imaging

In our double transgenic K5Tta × TRE-H2BGFP^+^ mouse model, nuclear GFP label is diluted in fast-dividing differentiated cells following administration of 2 g/Kg (Bio-Serv, Flemington, NJ, USA) dox in the mouse chow from 21 days (d) of age. In slow cycling cells (a characteristic of stem cells) GFP is retained allowing the tracing of these cells in the adult cornea. GFP^+^ mice corneas (n = 49) were examined under the slit lamp microscope at eye-lid opening stage and followed to adult hood (>6–8 weeks old) to ensure only mice with normal clear corneas were selected for our study. K5Tta × TRE-H2BGFP^−^ mice were also examined as controls (n = 20). Mice were administered dox at 21d old and sacrificed after 0, 10, 28, 53 & 91 d chase by CO_2_ asphyxiation. A subset of mice with ocular abnormalities (n = 22) exhibiting signs of neovascularization and conjunctivalization as a result of the genetic cross were sacrificed after 35, 42 and 49 d chase. Eyes were enucleated and the expression of GFP in corneal flatmounts was analyzed using a Zeiss Axiovision Observer fluorescence microscope.

### Corneal Epithelial Cell Isolation and Fluorescence Activated Cell Sorting (FACS)

The corneal epithelia from adult K5Tta × TRE-H2BGFP mice (with prior slit lamp screening for clear corneas) chased as described above at 28, 42 and 91 d were separated from the stroma by incubating entire globes at 4 °C overnight in CnT50 media (CELLnTEC, Bern, Switzerland) containing 12.6 units dispase (Roche Diagnostics, Basel, Switzerland) and 100 mM D-sorbitol (Sigma Aldrich, St. Louis, MO). The detached epithelial sheets were collected and incubated with 0.05% trypsin/0.03% EDTA (Life Technologies, NY) at 37 °C for 10 mins to isolate single cells using a spatula to detach corneal epithelium from the underlying corneal stroma. Enzymatic reactions stopped by adding media supplemented with fetal calf serum (FCS) (Sigma-Aldrich, St. Louis, MO) and washed by centrifuging several times. Cells were then washed with PBS twice and suspended in 1 ml of DAPI solution and strained through a 70 μm cell strainer. GFP^+^ and GFP^−^ cell populations were sorted using a FACSAria II (BD Biosciences, San Jose, CA) directly into tubes containing RNA lysis buffer (Qiagen, GmbH, Hilden, Germany) with 2-mercaptoethanol. 6–10 globes were used for each FACS procedure from clear and normal corneas at 28, 42 and 91 d chase and 14 globes for the abnormal corneas at 84 d chase.

### RNA isolation

Total RNA from FACS cells (GFP^+^ and GFP^−^ cell populations) was extracted using Qiagen RNeasy Plus Mini Kit (Qiagen, GmbH, Hilden, Germany), and RNA integrity and quantity were recorded using Agilent Technologies 2100 Bioanalyzer and Nanodrop Spectrophotometer at the Genomics Resources Core Facility of Weill Cornell Medical College.

### Next generation sequencing library preparation and Illumina sequencing

Total RNA from GFP^−^ and GFP^+^ corneal epithelial cells (minimum concentration of 100 ng) was provided to the Genomics Core Facility at Weill Cornell Medical College to create cDNA libraries (Illumina, San Diego, CA). Samples were multiplexed with 3 samples per lane and loaded onto flow cell lanes. Sequencing-by-synthesis of 58-nucleotide length, single-end reads were performed on Illumina Hiseq 2000/1000 sequencing system.

### Comparative RNA-Seq analysis from corneal epithelial GFP^+^ LRCs (slow cycling labeling retaining cells) and GFP^−^ (fast-dividing cells) from 28, 42, 84 and 91 days chase

Comparative analysis from clear corneas at 28, 42 and 91 d chase reads were aligned to the mm9 mouse transcripts using STAR (version 2.3.0e)^14^. Then HTSeq^15^ was applied to obtain gene counts, and multi-mapping reads were discarded according to suggested HTSeq practice. A total of 12597 genes were used for subsequent analysis after filtering genes with less than 20 average read counts of all samples. Gene count data was rescaled using DESeq2 (version 1.2.10) R package^16^. Samples were separated into “GFP^+^” and “GFP^−^” groups for each chase period, and then differentially expressed genes (DEG) were detected between the groups with a false discovery rate (FDR) < 0.05 and >2 fold change by using DESeq2. Venn diagrams summarizing the overlap of DEG among different chase periods were generated in R. Gene ontology (GO) and functional annotation was used for the enrichment analysis. The GO biological process terms were assigned to each gene in the list with MOSAIC (version 1.1)^17^, and then the gene lists for functional enrichment were analyzed by NOA (version 1.1)^18^. In order to control the type I error rate of multiple hypotheses testing, Benjianmini & Hochberg method was employed to adjust *P*-values, so GO terms were considered as statistically significant overrepresented functions with adjusted-*P* < 0.05. Besides the regular differential expression analysis for each individual chase period, we also evaluated the trend of gene expression changes between GFP^+^ and GFP^−^ over the chase time. A list of 3277 genes, differentially expressed in at least one chase period was obtained. From this list we picked 786 genes with monotonic fold changes over time (i.e. going from lower fold changes at 28 d chase to higher fold changes over chase periods in GFP^+^ and GFP^−^ cell populations). Sequence reads from GFP^+^ and GFP^−^ abnormal corneas were normalized with normal samples using DESeq2.

### Comparative analysis between corneal epithelial GFP^+^ cells and hair follicle stem cells

In order to obtain putative markers of corneal stem cells, we compared our data in the cornea from 42 and 91 d chase GFP^+^ LRCs to Cluster of Differentiation 34 (CD34^+^) HFSCs from WT back skin^13^ (GSE54424) and the data was analyzed in the same manner as above. A total number of 13150 genes were used for the comparison of corneal GFP^+^ cells from 42 and 91 d chase corneas versus HFSCs.

### Histological staining of adult cornea and conjunctival tissues

Adult 9 week old WT CD1 cornea and conjunctiva tissues were dissected in PBS, fixed in 4% paraformaldehyde (PFA) for 30 mins and embedded in Tissue Tek Optical Cutting Temperature compound (Sakura Finetek Japan Co., Tokyo, Japan) and snap frozen in liquid nitrogen. Cornea sections of 8μm were mounted onto Superfrost Plus Gold slides (Fisherscientific, MA) and stained with haemotoxylin and eosin.

### Immunofluorescence detection of RNA-Seq derived candidate genes

Adult WT CD1 6–10 week old globes were fixed in 4% paraformaldehyde (PFA) for 20 mins, corneas were dissected in PBS, embedded in Tissue Tek and snap frozen in liquid nitrogen. Cornea sections of 8 μm were mounted as above, washed in 0.1% PBST, blocked (10% normal goat serum (NGS), 1% Bovine Serum Albumin + 0.1% Triton × 100 in PBS (PBST)) for 1 hr at room temperature and primary antibodies were incubated overnight with rabbit polyclonal antibodies (in blocking buffer as above containing 5% NGS) Sry-Box 9 (Sox9) (Millipore, AB5535, 1:1000), Annexin A3 (Anxa3) (Proteintech, 11804-1-AP, 1:100), Frizzled 7 (Fzd7) (Millipore, 06–1063, 1:300), Actinin1 (Actn1) (Sigma, HPA006035 1:750), Keratin 17 (Krt17) (Santa Cruz, 101931, 1:250). The following day slides were washed twice in 0.1% PBST for 15 mins and an anti-rabbit Cy3 (Jackson Immunoresearch: 711-165-152, 1:500) was used as secondary antibody and incubated for 1 hr at room temperature in blocking buffer containing 5% NGS. Slides were mounted in Vectashield containing DAPI (Vector Laboratories, Inc., CA) for nuclear staining.

### Immuno co-localization of GFP^+^ cells with putative stem cell markers

Adult K5Tta × TRE-H2BGFP^+^ globes were fixed in 4% PFA for 4 mins prior to dissecting out the cornea in PBS to process for OCT embedding as described above. Corneal sections from chased corneas (at 35 and 53 d) containing strong nuclear localized GFP signals were used for co-localization of slow cycling cells with putative stem cell markers of the cornea. Sections were fixed using 1% PFA for 2 mins, blocked as above and co-stained H2B-GFP^+^ cells with antibodies Tumor protein 63 (ΔNp63) (Santa Cruz, sc8609, 1:200), Keratin 15 (Krt15) (Proteintech, 10137-1-AP, 1:200), Sox9 (Millipore, AB5535, 1:1000), Fzd7 (Millipore MA, 06-1063), Krt17 (Santa Cruz, sc101931, 1:250) and Actn1 (Sigma-Aldrich, HPA006035, 1:750).

### Periodic Acid Schiff (PAS) staining of corneal epithelium to confirm the absence of conjunctival goblet cells on the corneal surface

We required corneas devoid of vessels and goblet cells, as this is an indicator of conjunctivalization. The presence of blood vessels and goblet cells on the corneal surface was analyzed using slit-lamp images following PAS staining in WT and K5Tta × TRE-H2BGFP^+^ (0, 42, 56 and 91 d chase) mice. Frozen sections from corneas were cut at 8 μm and rehydrated prior to incubation in 0.5% periodic acid solution, followed by staining in Schiff (Electron Microscopy Sciences, PA) reagent. Slides were rinsed in tap water, immersed in Harris haemotoxylin, and further rinsed in water. Slides were dipped in 0.5% acid alcohol, rinsed in water, dipped in ammonium water and further rinsed in water. Sections were dehydrated in a graded series of alcohol, cleared in xylene and mounted with DePeX mounting medium.

### Bromodeoxyuridine (BrdU) pulse-chase co-labeling of H2BGFP^+^ cells from K5Tta × TRE-H2BGFP mice in the cornea

For label retention studies, 21 days old K5Tta × TRE-H2BGFP^+^ mice (n = 4) were injected intraperitoneally with 50 μg/g BrdU twice a day (at 12 hr intervals) for three consecutive days and fed with 0.8 mg/ml BrdU (BrdU, Sigma-Aldrich, St. Louis, MO) in water for three days. Mice were simultaneously chased with dox for 19 d and sacrificed. Globes were enucleated and the corneas were processed for sectioning (as described above). Frozen sections were cut at 7 μm, fixed in 2% PFA for 2 mins, washed in PBS and the sections were incubated in blocking buffer (as described above) containing DAPI for 30 mins at room temperature. The sections were washed in PBS and incubated in primary chicken polyclonal anti-GFP antibody (Abcam, ab13970, 1:1000) at 4 °C overnight. The following day, sections were washed in PBS with 0.1% PBST and were incubated with anti-chicken secondary antibody (Life technologies, A11039, 1:500) for 1 hour at room temperature. The sections were washed in PBST, fixed in 4% PFA for 15 mins, and further washed in PBS. Sections were incubated in 2 N HCL 37 °C for 30 mins, washed in PBS, blocked for 30 mins and incubated in anti-BrdU Biotin conjugated antibody (Millipore, MAB3262B, 1:100) at 4 °C overnight. The following day, secondary mouse antibody (Jackson Immunoresearch, 200-072-211, 1:150) was added in blocking buffer containing DAPI for 1 hour at room temperature. The sections were washed in PBS with Triton X 100, then incubated in PBS-DAPI for 2 mins, washed twice in PBST and then mounted in Vectashield (Vector Laboratories, Inc., CA).

### Microscopy

Carl Zeiss Axiovision Microscope (Carl Zeiss Microimaging GmbH, Jena, Germany) was used to observe corneal wholemounts. Images were taken with an Observer Z1 fluorescent microscope (Carl Zeiss, AG) at 10x and 20x objective lenses utilizing a 1.6 Optovar optic and mosaic tile images. AxioCam HRm digital camera (Zeiss) was used to capture phase contrast images. Immunofluorescence staining from corneal sections was captured using a Zeiss LSM-710 confocal microscope (Carl Zeiss, NY). Images were compiled in Photoshop CS5.1 and Illustrator CS5.1 (Adobe systems Incorporated, USA).

## Results

### The histone H2B-GFP pulse-chase system identifies slow cycling cells of the cornea

**Figure 1:**
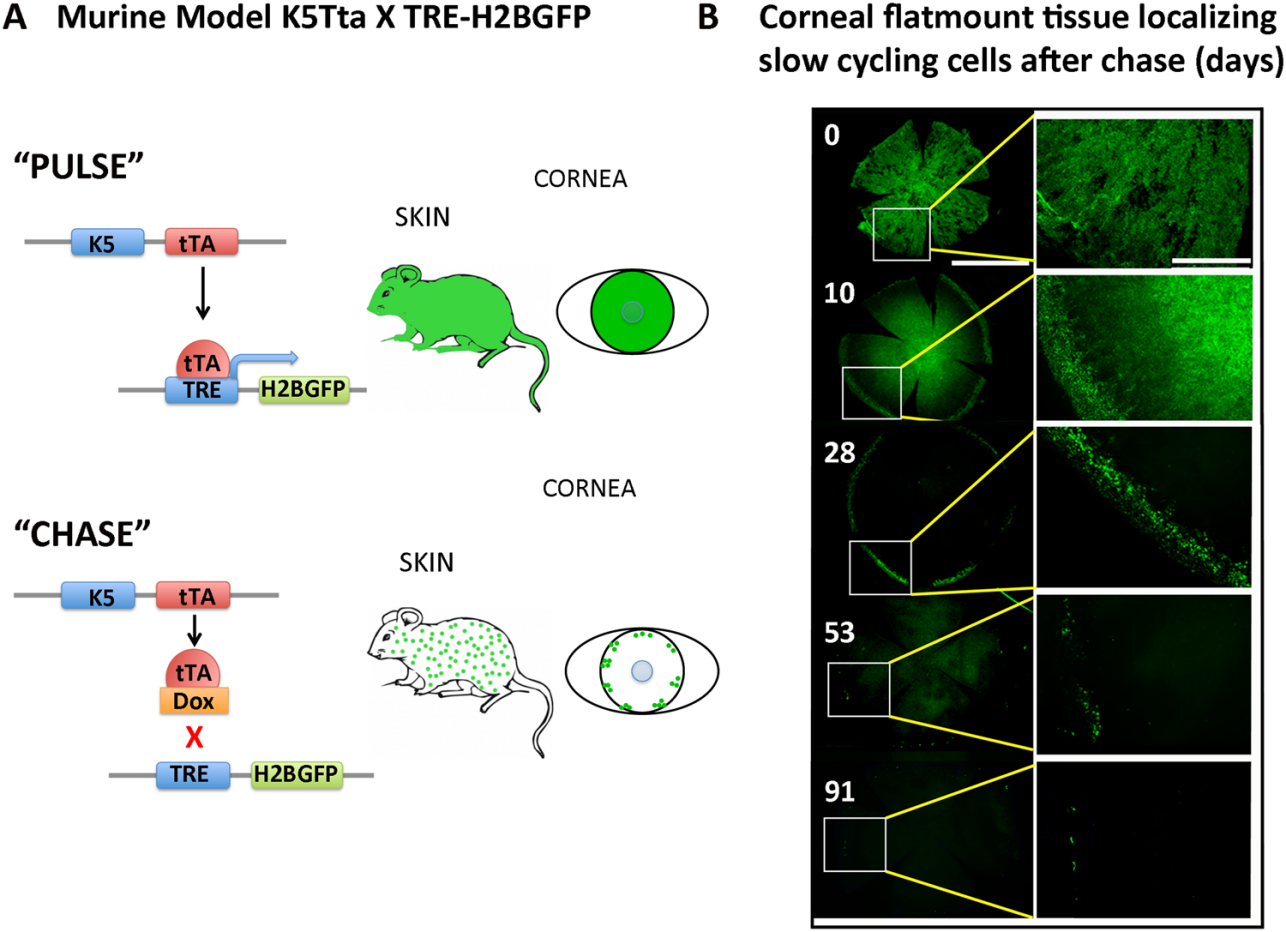
Murine K5Tta × TRE-H2BGFP identifies limbal localized GFP^+^ cells in corneal wholemount tissue. (**A**) Tetracycline-inducible (tet-off) double transgenic mouse system drives expression of histone H2B–GFP from the epithelial keratin 5 (K5) promoter (pulse). Dox administration in mouse diet turns off H2B–GFP expression (chase) when proliferating epithelial cells dilute the label between daughter cells by divisions. (**B**) Corneal flatmount tissue localizing slow cycling cell populations from K5tTA X TRE-H2BGFP mice starting from 0–91 d chase. Low magnification of the entire cornea is displayed on the left panel after 0 (n = 4), 10 (n = 2), 28 (n = 3), 53 (n = 5) and 91 (n = 3) d chase and higher magnifications from the boxed areas on the left panel are shown on the right. GFP^+^ nuclear staining marks the majority of the corneal epithelium at 0 d chase. At 10 d chase GFP positive nuclei are scattered throughout the cornea, with a distinct lack of GFP^+^ cells separating the periphery and the central cornea. Low magnification of 28 d chase shows few GFP^+^ cells in a  cluster localized to the periphery. The enlarged image at 28 d shows these GFP^+^ nuclei at the peripheral base and the limbal region. Low magnification of 53 d chase exhibits fewer cells in the periphery compared to 28 d chase, and the enlargement shows the limbal localization of these cells. At 91 d chase, GFP^+^ cells become few, but the location of GFP^+^ cells remain at the limbus as highlighted in the enlarged image on the right panel. Scale bar in B for right panel = 1000 µm and for the left panel images = 400 µm.

The double transgenic murine system has been used to detect slow cycling cells in various epithelial tissues^8,12,19,20^. The mating of two parent transgenic strains (K5Tta and TRE-H2BGFP) produces progeny in which the expression of a transgene encoding histone H2B-green fluorescent protein (H2B-GFP) can be turned off (beginning the chase period) when dox is added to the animal’s diet. (Fig. 1A). A time course analyzing the pattern of H2B GFP^+^ cells from 0–91 d chase enabled us to follow the label retaining cells in the corneal epithelium (Fig. 1B). At 0 d chase, GFP^+^ cells populated the majority of corneal cells on the ocular surface. After 10 d chase, a clear separation between the limbus and central cornea was observed, indicating the dox had taken effect by suppressing H2B expression. At 28 d chase, GFP expression was restricted to the corneal periphery and limbus. At the 53 d chase time point, GFP^+^ cells were few and localized only at the limbus, with a further diminution of GFP^+^ cells seen at the limbus by the 91 d chase time. These data suggested that the label-retaining cells were slow-cycling.

Unexpectedly, we found that some K5Tta × TREH2B-GFP^+^ mice spontaneously exhibit corneal anomalies as early as P14, including neovascularization, inflammation, and conjunctivalization (Supplementary Fig. 1). The corneas from adult WT FVB (Supplementary Fig. 1Aa), parent strain corneas K5Tta (Supplementary Fig. 1Ab) and TRE-H2BGFP (Supplementary Fig. 1Ac) were clear and normal when observed under slit lamp. We found an approximate 1:1 ratio of normal (Supplementary Fig. 1Ad), and abnormal corneas (as described above) (Supplementary Fig. 1Ae) in K5Tta × TRE-H2BGFP progeny. In all cases, corneas selected for molecular and histological analyses were devoid of corneal anomalies by pre-screening with slit lamp microscopy as seen in Supplementary Fig. 1A. In order to determine if outwardly “normal” mice had phenotypes that were left undetected by slit lamp microscopy, such as the appearance of goblet cells over the surface, we further analyzed these mice for signs of ocular surface abnormalities. Sections of PAS stained corneas from WT FVB, 21 d pulse or 0 d chase (Supplementary Fig. 1B), and pre-screened slit lamp imaged corneas from double transgenic mice after 42 and 91 d chase were devoid of goblet cells (Supplementary Fig. 1). However, PAS stained sections from mice with corneal defects (Supplementary Fig. 1Ad) revealed goblet cells at the surface of the cornea at 56 d chase (Supplementary Fig. 1). Additionally, differences in the distribution of GFP signal was observed when normal versus abnormal corneas were analyzed. In clear normal corneas from double transgenic mice chased for 28 d, a clear GFP^+^ ring was observed around the limbal region. Abnormal corneas from mice chased at 35, 42 and 49 d, showed a scattered distribution of GFP^+^ cells in limbal and central corneas with cellular appearance, and other parts were non-cellular and GFP^+^ due to autofluorescence, when viewed under fluorescent light (Supplementary Fig. 1C). We also observed that abnormal corneas have a different degree of differentiation when comparing the GFP^+^ cell population to GFP^−^ at 84 d to normal corneas from 91 d chase periods. In abnormal corneas, higher Krt12 (differentiation marker) expression was found in GFP^+^ cells (Supplementary Fig. 1D). As expected, 91 d chased GFP^+^ LRCs from normal corneas showed a significant decrease in Krt12 expression. Putative stem cell markers Tumor repressor protein (Trp63), Krt15, Fzd7, β-catenin (Ctnnb1), Sox9, Ifitm1 and candidate marker Actn1 all showed elevated expression in the abnormal GFP^−^ cell populations compared to the normal GFP^+^ cell populations found at 42 d chase (data not shown) and 91 d chase (Supplementary Fig. 1D). In contrast, Abcg2 was up-regulated in abnormal GFP^+^ cells compared to the GFP^−^ and was shown to have the opposite effect in our normal GFP^+^ LRCs (Supplementary Fig. 1D).

### Stem-like genes are significantly up-regulated as chase period’s increase

**Figure 2:**
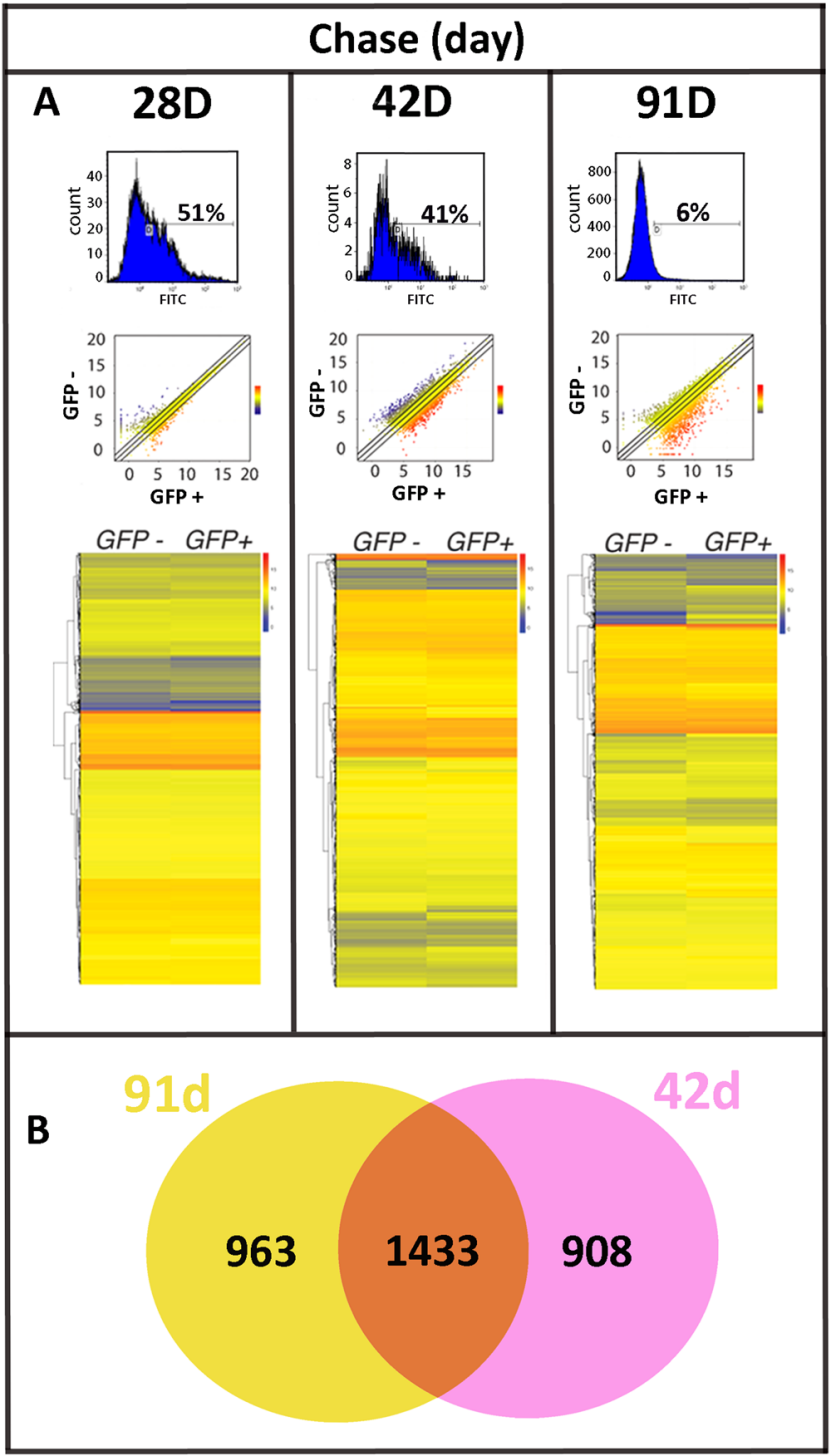
Differential gene expression between GFP^−^ and GFP^+^ corneal cells are greater at 42 d post chase and thereafter. (**A**) FACS analysis of freshly dissociated K5tTA X TRE-H2BGFP corneal epithelial cells demonstrated decreased GFP^+^ LRC percentages as the chase period was extended from 28 d (51%, n = 5) to 42 d (41%, n = 3) and to 91 d (6%, n = 5). The scatterplot of the RNA-Seq expression data shows the differences in gene expression between GFP^+^ (x-axis) and GFP^−^ (y-axis) in log2-transformed scale from 28 d, 42 d and 91 d chase. Gene expression change between GFP^−^ and GFP^+^ is represented as a color (blue, low expression to red, high expression). Differential gene expression increases at 42 d and 91 d chase. The heat map includes 12,597 genes for each chase period. Color differences between GFP^−^ and GFP^+^ become more apparent at 42 d chase onwards. (**B**) Venn diagram of significantly differentially expressed genes between GFP^−^ and GFP^+^ shows there are 1433 genes (orange) shared between 42 d and 91 d chase. There are 908 genes (pink) exclusively differentially expressed at 42 d and 963 genes (yellow) exclusively differentially expressed at 91 d chase.

To further characterize GFP^+^ LRCs in the cornea, we performed FACS analysis comparing them to GFP^−^ fast-dividing cells. The optimal chase duration was not known for the corneal epithelium. We examined three chase periods to ascertain the length of time needed to enrich for GFP^+^ cells expressing “stem-like” genes. FACS analysis at the 28 d chase demonstrated that 51% of corneal epithelial cells remained GFP^+^. By 42 d chase this decreased to 41% and then further reduced to 6% at 91 d chase (Fig. 2A). Negative control data using WT corneal epithelial cells from entire globes for each experiment were less than 1% GFP^+^ likely due to autofluorescence (Supplementary Fig. 2).

Scatterplots generated for differentially expressed genes for each chase period shows greater differences in gene expression in GFP^+^ LRCs as compared to the GFP^−^ population as chase periods increase (Fig. 2A). The heatmaps generated are visual representations of 12,597 genes, (red, high expression; blue, low expression) comparing the normalized read counts of each gene as a single line in GFP^−^ to GFP^+^ at 28 d, 42 d and 91 d chase with average read counts of less than twenty.

At 28 d chase, 487 (3.86%) genes show a significant change (2 fold change and FDR < 0.05) between GFP^+^ and GFP^−^ cells. At 42 d chase, this increased to 2341 (18.58%) genes, similar to the 2396 (18.80%) value obtained at 91 d chase. Of these genes, 247 were found to be significantly up-regulated in GFP^+^ cells at 28 d chase; 1207 at 42 d chase and 1515 at 91 d chase (Fig. 2). In contrast, 240, 934 and 881 genes were down regulated in chase period 28 d, 42 d and 91 d respectively.

The Venn diagram represents overlapping significant genes (2 fold change and FDR < 0.05) in 42 d and 91 d chase periods (Fig. 2B). The percentage difference of genes changed at 91 d chase to 42 d chase equates to 60%. The gene percentage shared between 42 d and 91 d is 61%. The data shows approximately 40% of genes unshared amongst longer time points of 42 d and 91 d chase (Fig. 2B).

### Gene ontology (GO) and pathway analysis for enriched genes in GFP^+^ LRCs and GFP^−^ differentiated cells

**Figure 3:**
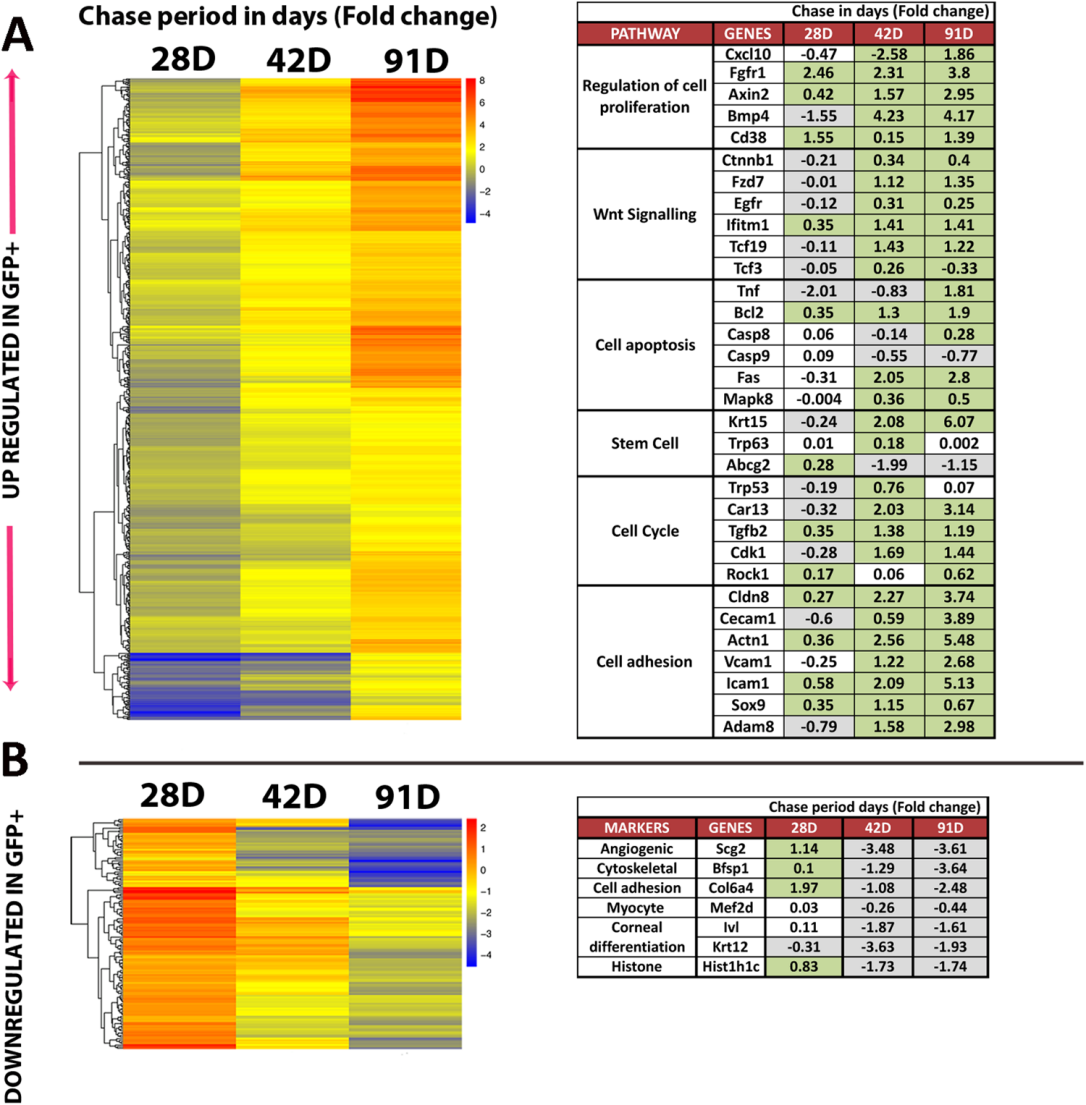
RNA-Seq analysis of GFP^+^ LRCs and GFP^−^ corneal epithelial cells from K5tTA × TRE-H2BGFP highlights the up-regulated and down-regulated genes in GFP^+^ LRCs over time. (**A**) Heatmap of 651 up regulated genes in GFP^+^ LRCs showing differences in gene expression between GFP^−^ and GFP^+^ cells represented in a single column at each chase period. The colors of the heat map representing single genes changing from blue to red across the chase periods indicate enrichment of gene expression in GFP^+^ LRCs. The table enlists pathways and genes involved in stem cell activation, maintenance and proliferation in GFP^+^ LRCs. (**B**) Heatmap of 135 down regulated genes in GFP^+^ LRCs. Blue represents low expression in GFP^+^, and red high expression in GFP^+^. The table indicates key genes involved in cell division and differentiation. Highlighted green boxes in the table represent significant high expression in GFP^+^ LRCs; Grey boxes represent significant high expression in GFP^−^ cells; No color indicates no significant difference between GFP^+^ and GFP^−^ cornea cells.

A total of 3277 genes demonstrated differential expression between GFP^−^ and GFP^+^ LRCs. We performed further analysis to determine if the changes in expression levels resulted in functional enrichment (Fig. 3). Using GO and KEGG (Kyoto encyclopedia of genes and genomes) pathway analysis, we found 786 genes with significant expression changes along the chase time points of 28, 42 and 91 d by the generalized linear model of DESeq2. This was then categorized into 2 enriched gene sets, 1) 651 up-regulated in GFP^+^ LRCs ascending the chase periods (Fig. 3A) and 2) 135 down-regulated in GFP^+^ LRCs, descending expression across chase periods (Fig. 3B). The most common trend for regulation of genes in these enriched sets was low expression difference (blue) between GFP^+^ and GFP^−^ LRCs at 28 d chase with subsequent high expression difference (red) at 91 d chase. In addition to genes identified by our statistical analysis, we also included a table of genes implicated in corneal epithelial maintenance (Fig. 3A). Here, we found an elevated expression of Krt15, Sox9, Actn1, Fzd7, Bone morphogenic protein 4 (Bmp4), Transcription factor 3 (Tcf3), Interferon Induced Transmembrane Protein 1 (Ifitm1) and C-X-C motif chemokine 10 (Cxcl10) in GFP^+^ LRCs. Genes enriched in GFP^−^ cells included epithelial differentiation marker Krt12 and Ivl. We also show that these cells significantly include an enriched level of the histone gene Histone cluster 1 H1 family member c (Hist1h1c).

### Hair follicle stem cell markers are shared by GFP^+^ LRCs of the cornea

**Figure 4:**
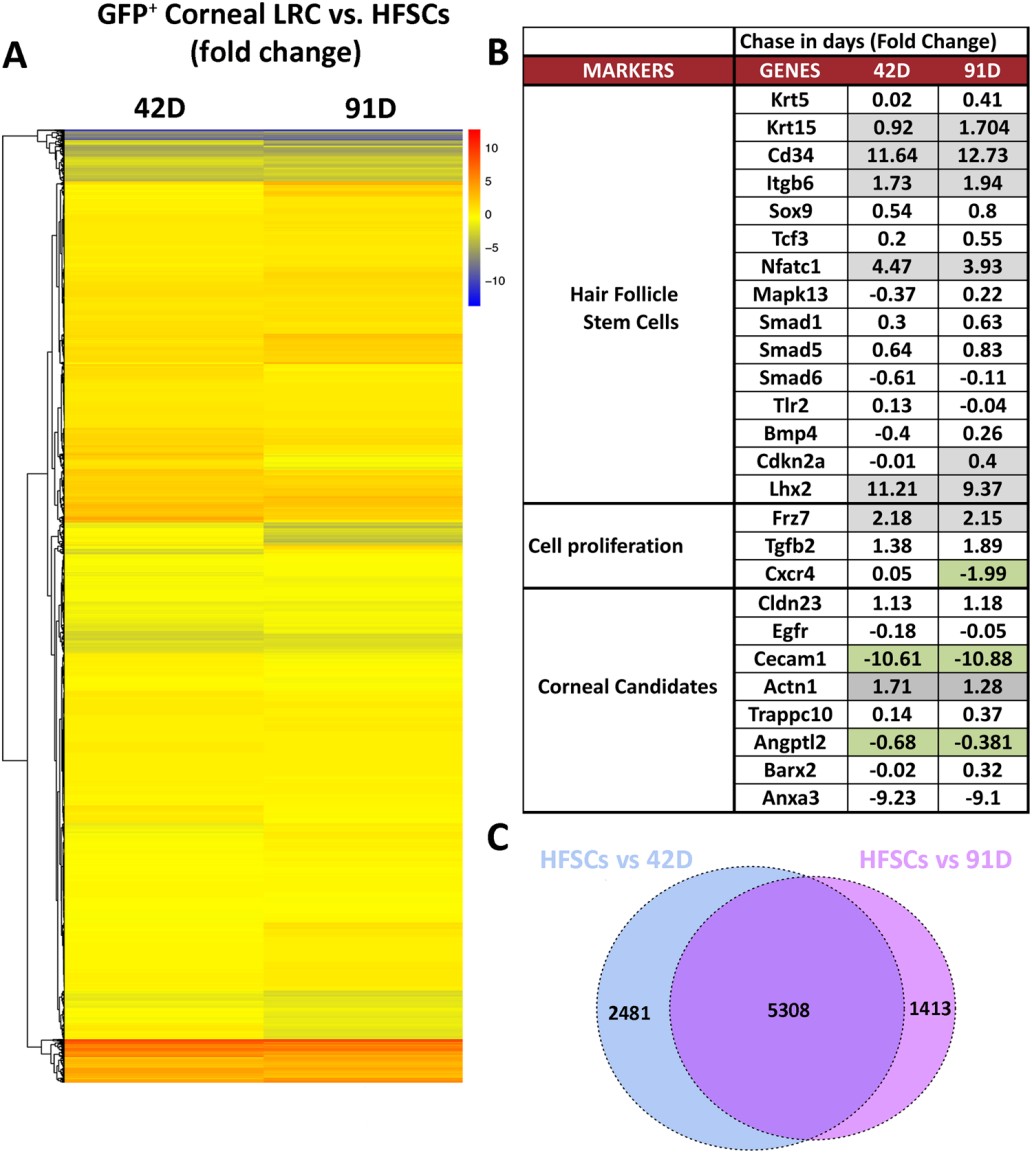
RNA-Seq analysis between hair follicle stem cells (HFSCs) and corneal GFP^+^ LRCs show markedly high similarity in gene expression. (**A**) Heatmap comparing RNA-Seq expression of GFP^+^ LRCs at 42 d chase (left, n = 3) and 91 d chase (right, n = 5) to CD34^+^ HFSCs gene expression (Kadaja *et al*.^13^). Blue indicates lowest expression while red means high expression in GFP^+^ cornea LRCs. The heatmap includes 9202 genes showing a high similarity in gene expression between the corneal GFP^+^ LRCs versus HFSCs as indicated by the lack of color change. (**B**) Comparison of expression levels for genes involved in HFSC stemness, cell proliferation and corneal candidates. Boxes highlighted in green indicates significant expression in GFP^+^ LRCs of the cornea; those highlighted in grey, show significance expression in the skin and no color indicates no significance difference between HFSCs and GFP^+^ LRC selected genes. (**C**) Venn diagram showing 5308 genes with similar expression levels between HFSCs and GFP^+^ LRCs of the cornea at 42 and 91 d chase periods. The diagram also shows that 2481 genes are differentially expressed between HFSCs and 42 d chase (blue) and 1413 genes are solely differentially expressed between HFSCs and 91 d chase (pink).

We compared the gene expression of WT CD34^+^ HFSCs (data obtained from Kadaja *et al*.^13^) to LRCs from the corneal epithelium at 42 d and 91 d chase (Fig. 4). Later chase periods were selected since in post 42 d chase the molecular analysis shows increased regulation of several stem cell related genes (as seen in Fig. 2). As discussed above, many regulated genes were common to both chase periods, but each period had a set of distinctly regulated genes, as well. The heat map represents the differences in gene expression between CD34^+^ HFSCs and GFP^+^ LRCs at 42 and 91 d chase respectively. Similar trends are observed across the two panels since the colors displayed little variation (Fig. 4A). A comparison of genes expressed in HFSC markers to the GFP^+^ LRCs at 42 and 91 d chase are highlighted in a table (Fig. 4B). In this analysis we found that the same level of expression of HFSC markers Krt5, Sox9, Tcf3, Contraction of Sma and Mad (Mothers against decapentaplegic) (Smad) 1, 5, 6, Trl2 and Bmp4, was found in corneal GFP^+^ LRCs (42 and 91 d chase). CD34 was found to be specific to HFSCs due to negligible expression in corneal epithelium. In addition, we pooled novel corneal candidates from our comparison to HFSCs to test on corneal tissue that were 1) Not significantly different between HFSCs and LRCs of the cornea; 2) Significantly up-regulated in only the HFSCs; 3) Significantly up-regulated in corneal epithelial LRCs at post 42 d chase compared to HFSCs. Collectively, these genes are known to play a role in maintaining epithelial HFSCs (Fig. 4B).

The Venn diagram shows differential expression analysis between CD34^+^ HFSCs and GFP^+^ LRCs from 42 and 91 d chase respectively (Fig. 4C). There are approximately 1000 more differentially expressed genes between HFSCs and 91 d chase compared to 42 d chase. There are 5308 genes shared between HFSCs and cornea LRCs at 42 and 91 d chase from a total of 13150 genes (Fig. 4C).

### GFP^+^ LRCs co-localize with putative stem cell markers

Protein expression of candidate genes identified from the differentially expressed GFP^+^ versus GFP^−^ corneal epithelial cells from RNA-Seq analysis was first determined by immunolocalization on wild-type corneal sections (shown in Supplementary Fig. 3). Immunocharacterization of Sox9, Krt17, Anxa3, Fzd7 and Actn1 (red) merged with DAPI (nuclear staining) are shown on sections of WT corneas (Supplementary Fig. 3).

**Figure 5:**
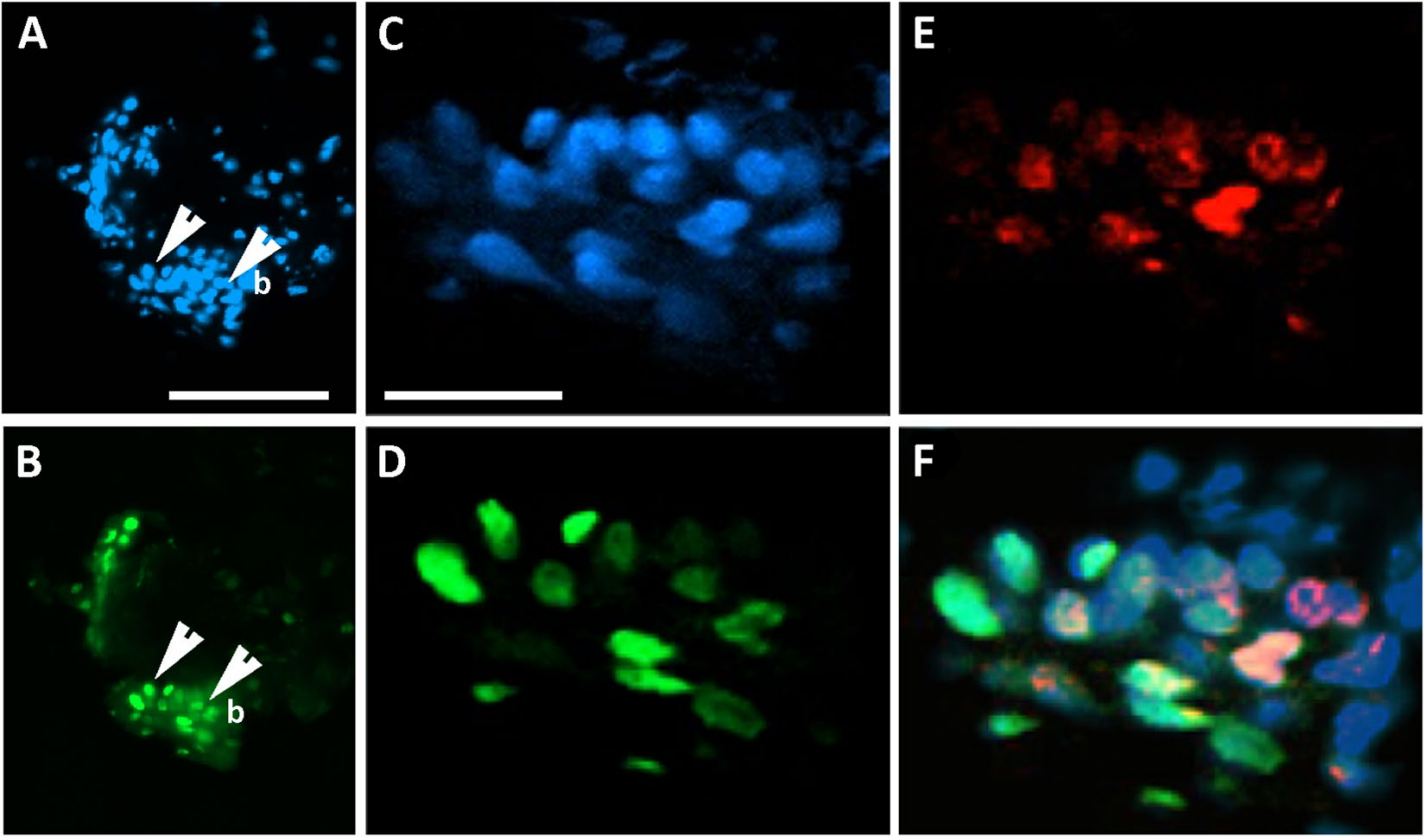
Pulse-chase co-labeling of H2B-GFP and BrdU at the limbus identify slow cycling and mitotically dividing cells. K5Tta × TRE-H2BGFP^+^ mice at 21d old were injected with BrdU and fed with dox for 19 d as described in the Methods. (**A**) DAPI nuclear staining of the corneal limbal region co-localizes with (**B**) H2B-GFP^+^ expressing cells. Higher magnification of the limbus shows detailed (**C**) DAPI nuclear staining that co-localize with (**D**) H2B-GFP and (**E**) BrdU. (**F**) The merged image shows co-localization of a group of H2B-GFP^+^ cells with BrdU at the limbus indicating that some cells are still dividing after the chase period. b, basal layer; white arrows in A&B point to the basal cells of the cornea, enlarged in C − F respectively. Scale bar A − B = 100 μm, C − F = 25 μm.

BrdU co-localization with H2B-GFP^+^ cells marked approximately 80% of cells at the limbus at 19 d chase (Fig. 5). A mixed population of bright, medium and low expressing H2B-GFP^+^ cells were detected, with greater numbers of GFP^+^ cells marking the basal layer and weaker H2B-GFP^+^ expression extended to a few cells in the suprabasal layers of the limbus (Fig. 5B,D). The data shows that after this chase period, the BrdU^+^ cells co-localized with H2B-GFP^+^ cells with high and medium expression at the limbus as cells were dividing towards the suprabasal layers (Fig. 5F).

**Figure 6:**
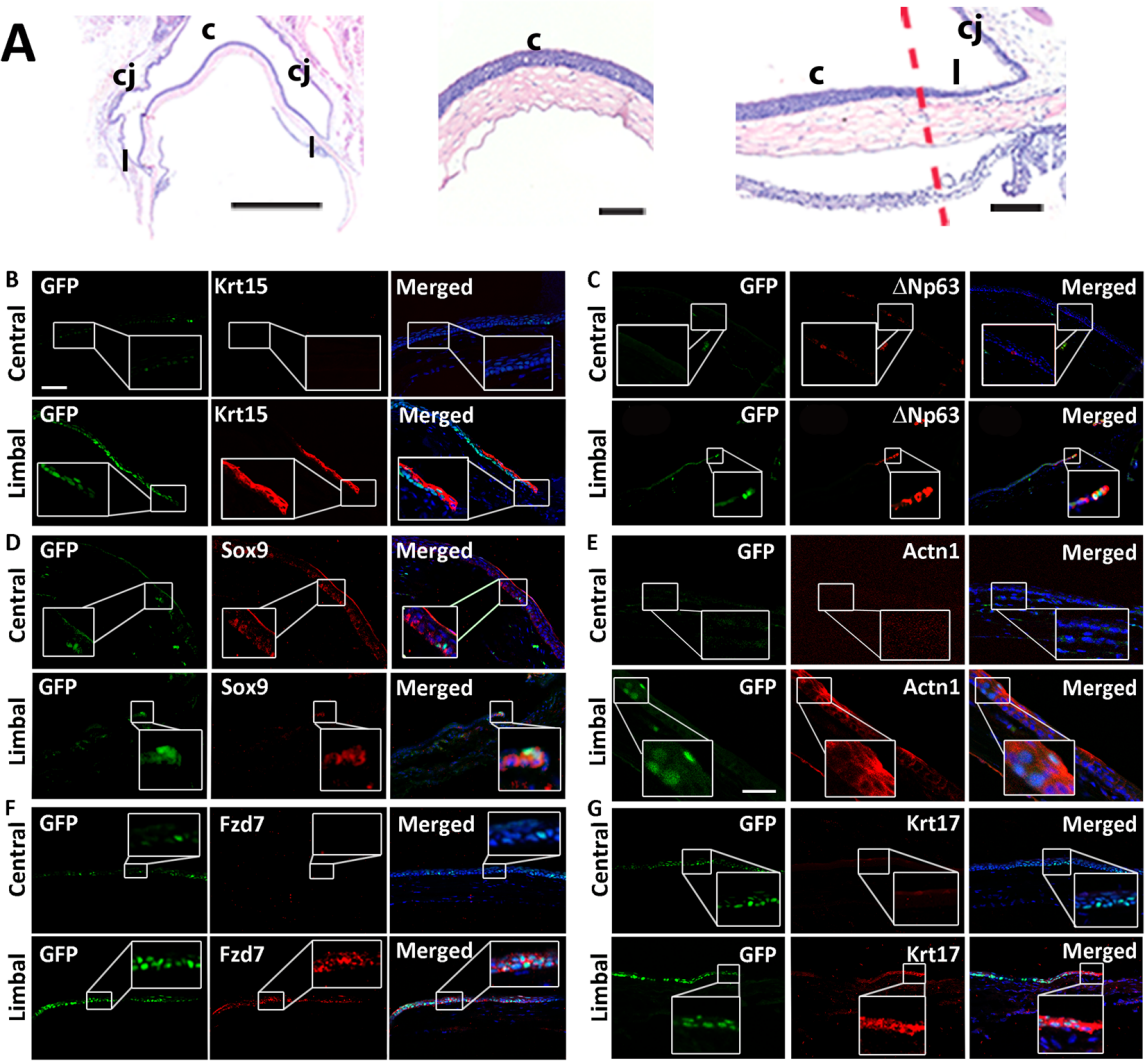
K5Tta × TRE-H2BGFP^+^ slow cycling cells co-localize with corneal stem cell markers. (**A**) Low power haemotoxylin and eosin staining of the adult cornea, limbus and conjunctiva tissue (left), magnified images of the central cornea (middle) and limbal cornea (right). (**B**–**G**) Corneal immunofluorescence staining of K5Tta × TRE-H2BGFP sections, co-stained with putative stem cell markers in the central (upper panels) and limbal cornea (lower panels). (**B**) At 35 day chase (n = 3) few lowly expressed GFP^+^ cells were found in the central cornea while Krt15 (red) antibody staining is not expressed. At the limbus, there is high GFP^+^ LRC staining at the limbal and periphery basal layer, including to one cell layer above in some areas with weaker expression. Krt15 and few GFP^+^ cells co-localize in limbal cells as shown in the merged panel. (**C**) In the central cornea, GFP^+^ LRCs at 53 d chase (n = 3) and ΔNp63 (red) labeled staining was absent. At the limbus, GFP^+^ LRCs were localized to 2–3 cells at the limbus and ΔNp63 expression was limbal and basal specific (nuclear expression). The merged image shows the co-localization of GFP and ΔNp63. (**D**) GFP^+^ LRCs at 53 d chase localized to a few cells in the central cornea (boxed) while Sox9 (red) marked only basal epithelial cells (n = 6). The merged image co-localizes them together. At the limbus, GFP^+^ LRCs co-localized with Sox9. (**E**) GFP^+^ LRCs from 53 d chase were not detected and Actn1 was devoid of staining in the central cornea. At the limbus, few GFP^+^ cells were visible and co-localized to cytoplasmic Actn1 staining (n = 4). (**F**) GFP^+^ LRCs cells at 35 d chase are strongly expressed in the basal and some suprabasal layers of the central cornea, while some weak staining was also observed in cells of the suprabasal layer. Fzd7 staining in the central cornea is devoid of any expression, while at the limbus, the expression is basal and co-localized to GFP^+^ LRCs (n = 2) (**G**) GFP^+^ LRCs are strongly expressed in the basal and some suprabasal layers of the central cornea, while some weak staining was also observed in cells of the suprabasal layer. Krt17 staining was absent in the central cornea, but co-localized with GFP^+^ LRCs at the limbus (n = 3). Scale bar in A = 200 µm for (**A**–**C**,**E**,**F**); in D scale bar = 50 µm.

To further implicate candidate genes in the maintenance of the LRC population, co-localization analysis was performed on K5Tta × TRE-H2BGFP corneal tissue for candidate and putative stem cell markers (red) with LRCs (green) at 35 d chase (Krt15, Krt17 and Fzd7) and 53 d chase (P63, Sox9 and Actn1) (Fig. 6). Histological staining of the entire globe without the lens tissue in low power included the central cornea, cornea-limbal and corneal-conjunctival tissue. Higher power images indicate the central (more differentiated) and the limbal (stem cell rich) regions of the cornea (Fig. 6A). At 35 d chase, H2BGFP^+^ cells were still expressed in a high (basal), medium (suprabasal) and low (suprabasal) manner, while at 53 d chase, H2BGFP^+^ cells were peripherally localized and mostly basal. GFP^+^ LRCs after 35 d chase, marked the basal cells at the peripheral cornea and limbus. Stem cell marker Krt15 was not found in the central cornea and only localized at the limbus. The co-localization of few GFP^+^ LRCs at the limbus with Krt15 was observed (Fig. 6A). GFP^+^ LRCs at 53 d chase were localized to few cells in basal limbus cells only co-localizing with the majority of ΔNp63+ limbal basal cells (Fig. 6B). Epithelial stem cell marker Sox9 co-localized with GFP^+^ cells at the limbus, and the expression extended throughout the basal cells of the cornea (Fig. 6C). Actn1 staining in the central cornea marked approximately half of the central corneal basal cells (see Supplementary Fig. 3C), with greater expression at the limbus, co-localizing with few GFP^+^ LRCs (Fig. 6D). At 35 d chase, Fzd7 extended the limbus and was devoid of expression in the central cornea. Fzd7 co-localized with GFP^+^ cells at the limbus that were basal including H2BGFP^+^ cells included some cells to a row above (Fig. 6F). Krt17 co-localized with GFP^+^ cells at the limbus, in particular the basal cells (Fig. 6G).

## Discussion

Limbal stem cell (LSC) isolation, and subsequent use of these purified stem cells for possible therapeutics has proven to be challenging. Strategies to purify LSCs could include the use of validated stem cells markers coupled with FACS. However, this strategy, largely effective for stem cells of other lineages including HFSCs^21^, hematopoietic and mesenchymal stem cells^22,23^ is not so straightforward for corneal LSCs given the paucity of bona fide LSC markers. A second strategy, using the underlying cell biological properties would not rely on prior knowledge of stem cell markers, but would instead be agnostic to previously defined proteins or molecular markers. An important characteristic of LSCs, and indeed stem cells in general is their slow cycling phenotype. Slow cycling stem cells reported in the salivary glands^11^ sebaceous glands^10^ and hair follicles^7^ reside in “niches”, dividing to produce daughter stem cells. Despite technical advances made to label slow cycling LSCs using tritiated thymidine or BrdU, they cannot be purified for molecular characterization, hence a mouse genetics approach labeled their slow cycling nature with GFP^2^.

Prior to dox administration in our model, GFP^+^ cells migrate centripetally over the ocular surface forming stripes and spiral patterns consistent with other studies when LacZ or fluorescently labeled transgenics are made^12,24–26^. Soon after dox administration and the chase begun, we observed that the extensive GFP labeling changed to specific regions of highly GFP positive cells, one at the limbus, and the other in the center of the cornea, forming a “doughnut-like” configuration. While both regions represent populations of non-dividing cells, the loss of this central labeled population as the chase periods were extended suggests that the labeled cells at the limbus represent a true slowly dividing stem cell compartment while the cells in the center merely represented terminally differentiated corneal epithelial cells which were sloughed from the surface over time. A clear stream of GFP^+^ dividing cells migrated towards the center suggesting that the source of cell renewal is from the limbus as previously reported^2,27,28^. In addition to the spatial localization in the X-Y plane to the limbus, we also saw localization in the Z plane as the chase periods lengthened. The observation seen in GFP^+^ dividing cells followed X, Y, Z hypotheses of Thoft, R. A. & Friend, J., in which stem cell mobility and migration derives from limbal cells at the periphery that divide, giving rise to transient amplifying cells, moving centripetally through the basal epithelium undergoing a limited number of divisions on the central cornea^29^.

After long-term dox administration >53 d chase, GFP^+^ cells were limbal specific, residing in a “niche”. GFP^+^ distribution throughout the corneal epithelial layers in early dox administration was prominently green and cells were sloughing off as part of the natural corneal renewal process. After long-term dox administration, GFP^+^ cells were switched off in (fast-dividing) supra-basal layers and LRCs were basally restricted at the limbus. This prompted us to further characterize these cells through FACS and high throughput sequencing.

The initial characterization of the transgenic mice demonstrated that approximately 50% presented corneal anomalies including signs of conjunctivilization and vascularization. We compared RNA-Seq data from abnormal and normal corneas and found that the tissues from abnormal corneas present a different subset of gene expression likely due to signs of vascularization, conjunctivilized overgrowth and other unknown anomalies compared to the normal clear corneas of the same transgenic mice. These side effects could be due to the deleterious effects of GFP, since several studies have shown that high levels of GFP can be toxic to cells *in vivo* in transgenic animals^30,31^. In half of the pups born, anomalies were detected at eyelid opening suggesting that GFP toxicity to cells may have occurred in the embryo. In our studies the phenotypes were observed in both eyes of an affected animal. However, we did note in rare cases that only a single eye has the abnormal phenotypes described at the time of eyelid opening, but that these mice ultimately are affected. This would favor an explanation of these abnormalities based on genetic background and not a stochastic effect. We are unsure of the exact molecular mechanisms that have resulted in the corneal phenotypes seen in these mice, however in our studies we excluded mice with abnormal corneal phenotype at eyelid opening and once again prior to obtaining their corneal tissue for the experimental procedures in this paper.

Dox administration commenced at 21 d old, prior to when the stem cells are suggested to fully reside limbally at around 4–5 weeks old^26,32,33^. Switching off GFP in actively dividing cells at this time period ensured GFP label retaining in slow cycling cells for long periods, over 100 d chase. Initially, we were not certain of an exact chase period in mice to obtain slow cycling cell populations. Therefore, we started chase at 21 d old ensuring the animals were not too old when chasing for extended periods. LESC holoclone production efficiency in human corneas have known to decrease with age^26,34^. In rat and mice corneas stem cells were localized throughout the ocular surface in basal cells up to two weeks post birth^26,33,35^. Then, preferential binding of the stem cell marker occurred at the limbus after two weeks of age for mice and after 4 weeks for rats^35^. Although the exact age at which LSC appear in mice is undetermined, studies have shown the postnatal loss of stem cells from the central cornea using analysis of mosaic mouse corneas show LSC maintenance occurs between 5–8 weeks^32^. Similarly, with increased age, the corrected number of radial stripes in the corneal epithelium declines from ~100 at 10 weeks age to ~50 at 39 weeks, with no further decline up to 52 weeks^32,36^. The number of active LESCs not necessarily decline with age, but there is a reduction in the number of LESC clones.

The first appearance of entirely peripheral GFP^+^ LRCs in the cornea was observed at 28 d chase, however, the appropriate chase period to isolate true LSCs by FACS may not coincide merely with limbal localization, but instead may also require the enriched expression of stem cell genes occurring at later chase periods. We combined our H2B-GFP localization with results from molecular characterization of cells purified from GFP^+^ cells at increasingly longer chase time points to define an appropriate chase period. Over time, GFP expression at the limbus became sparse, suggesting that further enrichment for the slowest cycling cells beyond 28 d chase was required to identify LSCs. Later, molecular changes in GFP^+^ cells at the limbus, isolated at 28 d, 42 d and 91 d chase were compared.

It was evident in our analysis that 42 d and 91 d chase shared a greater percentage of gene similarities than either did with 28 d chase. That being said, the significantly up-regulated genes at 42 d and 91 d chase expressed unique markers at each chase period, suggesting that GFP^+^ LRCs represent different subsets of cells with increased chase time. At 28 d chase, the heat map generated in GFP^−^ and GFP^+^ populations did not produce as many differential genes as the genes expressed between the two populations at 42 d chase onwards. At 42 d chase we detected significantly up-regulated landmark genes in our LRC population such as P63, Krt15 and Sox9. Although the RNA-Seq expression of GFP^+^ LRCs presented at 42 d and 91 d chase shared approximately 60% of genes respectively, each cell group represented unique slow cycling cell populations with 40% of genes that were not shared. These differences were likely due to increased aging in animals as chase time progressed and the selection for slower cycling cells with increased chase periods.

In general, cell labeling protocols of the cornea have used a chase period of 56 days to decipher limbal localization of the slow cycling cells^2,12,37,38^. Previous reports have shown that there was between 0.92–3.6% of LRCs in mouse corneas labeled with BrdU at P3 and chased till adulthood (6–8 weeks), with a preference of slow cycling cells at the inferior and superior quadrants^37^. In our data, at the 42 d chase period, there were greater numbers of GFP^+^ cells compared to 91 d chase. We would expect a mixed population of actively dividing and quiescent cells to be expressed at 42 d chase and a likely increase in the proportion of cells that were slow cycling at 91 d chase. ﻿After BrDU and doxycycline administration after 19 d chase in our double transgenic mice, H2B-GFP^+^ cells co-localized to BrDU^+^ cells at the limbus. ﻿Corneal epithelial cell turnover time in mice (time taken for a basal cell to travel to the surface and slough off) is less than or equal to 14 days as tested by BrdU labeling^39,40^. The turnover time is shorter than the time required for LSCs to replace the entire corneal epithelium, which is estimated to last 7 weeks in mice^39^. Our double labeling (BrdU and H2B-GFP) of the limbal tissue at 19 d chase, demonstrated that limbal cells are co-labeled effectively at a period where corneal epithelial cells are both in mitotic and slow dividing states.

It has been shown that HFSCs retain GFP labeling at the bulge up until 10 weeks following chase, whereas LRCs in the epithelium from the anal to rectal transition zone showed almost no GFP^+^ cells after 5 weeks of chase^41^. Therefore, ideal chase periods identifying slow cycling cells vary with different tissues.

Stem cell populations are both actively proliferating and quiescent. Our model is designed to target LRCs at G0 (quiescent phase). Quantitative analysis from previous reports has shown no evidence of a decline in LRC numbers between 15 and 30 weeks old in WT mice^39^. Since our chase times varied, the mice ages from each chase period also varied from 49, 63 and 112 d old suggesting that our chase time of 91 d (16 week old), although prolonged, may not affect the number of LRCs over time.

Comparative microarray expression of genes in human central and limbal corneas identified four genes, namely Krt15, Krt14, Cdh3 and Wnt4 as limbal and basal rich, and significantly up-regulated in corneal stem cells^42^. These markers were then co-localized with BrdU labeling in human limbal explants showing that they are slow cycling in nature^42^. Our data in mice also showed that these four markers mentioned above were significantly up-regulated in GFP^+^ LRCs at 42 d chase and onwards.

To corroborate the RNA-Seq expression in this model with known molecular markers of stem cells in the literature, we looked at genes up-regulated in LRCs of the cornea including the Wnt signaling gene network. Products of these Wnt genes act as niche factors maintaining cell renewal^43–45^ by regulating cell proliferation and binding to Frizzled (Fzd) cell surface receptors. Fzd7 belonging to the Frizzled family of G-coupled receptors mark limbal basal cells^46^ and was significantly up-regulated at the limbus compared to the central cornea. ß-catenin controlling the stability of Fzd, (also up-regulated in GFP^+^ LRCs after 42 d chase) functions with the protein transcription factor 3 (Tcf3)^47^ not yet reported in the corneal epithelium regulating cell cycle progression. Fzd7 and Tcf3, known as epidermal stem cell markers were up-regulated at 42 d chase onwards, but not at 28 d. Similar increased expression of the stem cell related genes interferon induced transmembrane protein 1 (Ifitm1, a marker significantly up-regulated in LSCs^48^) and Keratin 15 (Krt15, an epithelial stem and progenitor marker^49,50^) were also observed with Ifitm1 expression and Krt15 expression up-regulated at in GFP^+^ LRCs.

Cell adhesion molecules (CAMs) known to specialize in cellular niches protecting and maintaining stem cells^51^ were observed in GFP^+^ LRCs. Actinin 1 or α-Actinin (Actn1) expression has previously been reported in the cornea (embryonic chick^52^ and rat^53^). In our experiments, Actn1 was significantly up-regulated throughout chase periods in GFP^+^ LRCs. Likewise, Sry box 9 (Sox9), a cell adhesion molecule and an epithelial stem cell marker^13,54,55^ was up-regulated in all chase periods in GFP^+^ LRCs. We examined cell cycle related genes changes between slow cycling cells and their faster dividing counterparts. Quiescent cells in the cell cycle exit at G0 stage, where they do not replicate. Our RNA-Seq analysis examined genes involved in cell division that included the Cyclin dependent kinases (Cdks). These genes act as key regulators of the cell division cycle and we found a significant elevation of Cdk1 in GFP^+^ cells at 42 d chase and thereafter suggesting a decrease in cell division. In contrast, the expression of known differentiation markers such as Keratin 12 (Krt12) and Involucrin (Ivl) were significantly increased in GFP^−^ cells, as might be expected. The Histone gene, Hist1h1c1 was enriched in our GFP^−^ populations after 42 d chase suggesting the presence of fast-dividing cells. No other histone complexes were significantly observed in our LRC populations after 42 d chase. Surprisingly, in our corneal RNA-Seq analysis we did not identify ATP binding cassette subfamily B member 5 (Abcb5), a more recent corneal stem cell marker^33^ expressed in either GFP^+^ or GFP^−^ cell population.

The most promising limbal stem cell markers in humans such as, CCAAT enhancer-binding protein δ (C/EBPδ)^56^, BMI1 Proto-Oncogene, Polycomb Ring Finger (Bmi1)^56^, ΔNp63α^57^, ΔNp63^57,58^, ATP-binding cassette sub-family G member 2 (ABCG2)^1,59^, N-Cadherin^60^ (N-cad or Cdh2), and Integrin α9 (ITGA9)^59^ localized in basal cells. Our data in mice has corroborated the expression pattern of human ΔNp63 to being limbal and basal specific. However, neither Itga9 (detected) nor Cdh2 (undetected) were significantly up-regulated in GFP^+^ LRCs at any chase period. Abcg2 and C/ebpδ were only up-regulated at the 28 d chase period.

The cornea, like the epidermis, functions as a barrier, playing a critical role in maintaining the immune-privileged status of the cornea, its clarity, and wound healing properties. Our data comparing cornea versus skin (data obtained by Kadaja *et al*.^13^) showed similar expression of HFSC markers Krt15, Sox9, Tcf3, Smad1, 5^61^, 6 and Fzd7 to corneal derived LRCs perhaps suggesting analogous functions in these cell types. Given the differences in distribution of stem cells (bulge niches across skin and specific limbal niches for the cornea) as well as differences in the proportion of these epithelia (keratinized skin vs non keratinized cornea) we would expect some possible differences in the gene expression of stem cells in these tissues. As expected we found the HFSC marker CD34 was unique to skin and barely expressed in the cornea, and we also found Nuclear factor of activated T-cells, cytoplasmic 1 (Nfatc1) expression, significantly expressed in HFSCs.

We tested our skin-cornea analysis by looking in WT cornea tissue for the presence of highly up-regulated stem cell markers using immunostaining. We found high expression of Krt15, Fzd7 and Krt17 in limbal basal cells, whilst Sox9, Actn1 (cytoskeletal protein) and Anxa3 (membrane and cytoplasmic), expression also extended to few basal epithelial progenitors, thus corroborating our RNA-Seq data.

Localizing GFP^+^ cells in our model after longer chase periods were difficult to obtain since fewer GFP^+^ cells exist through the sections. Therefore, we used chase periods under 56 d chase to co-localize GFP^+^ LRCs to candidate markers. We explored the expression of stem cell markers in the cornea Krt15, ΔNp63 and Sox9 by co-localizing this gene to GFP^+^ LRCs in the nuclei of our corneas from the double transgenic. These markers although significantly up-regulated at the limbus, were not specific to slow cycling cells. Similarly, Actn1 expression was not exclusive to GFP^+^ LRCs and was prominently expressed in basal limbal cells extending to 50% of the central basal cells. In other tissues, this gene regulates cytokinesis, cell adhesion, spreading, migration and signalling^62^. From our pathway analysis we showed that 28 d chase may not be sufficient time for slow cycling cells to exist, since many of the key regulators involved in stemness namely, Krt15, Bmp4, Fzd7, Ctnnb1, Carcinoembryonic antigen-related cell adhesion molecule 1 (Cecam1) and A Disintegrin and metalloproteinase domain-containing protein 8 (Adam8) were not significantly up-regulated in the earlier chase stage. Given the expression of putative stem-like genes presented in our RNA-Seq results from GFP^+^ LRCs and GFP^−^ cells, slow cycling cells would be best obtained after 42 d chase, closer to 91 d chase periods.

Our model isolates slow cycling cell populations of the corneal epithelium at the limbus but in greater quantities than we expected. Label retaining cells using this model has proven useful in studying corneal epithelial cell turnover to monitor divisions over time. This system can label virtually all LSCs independent of their cell cyclic stage and the gene expression analysis has provided an array of molecular markers showcasing key differences between the “fast” and “slow” cycling cells.

## Electronic supplementary material

Characterization of slow cycling corneal limbal epithelial cells identifies putative stem cell markers.
