# Author Correction: Characterization of slow cycling corneal limbal epithelial cells identifies putative stem cell markers

**DOI:** 10.1038/s41598-018-20584-x

**Published:** 2018-02-01

**Authors:** R. Sartaj, C. Zhang, P. Wan, Z. Pasha, V. Guaiquil, A. Liu, J. Liu, Y. Luo, E. Fuchs, M. I. Rosenblatt

**Affiliations:** 1 University of Illinois, Chicago, USA; 2 Weill Cornell Medical College, New York, USA; 3 The Rockefeller University, New York, USA

Correction to: *Scientific Reports* 10.1038/s41598-017-04006-y, published online 19 June 2017

The incorrect version of the Supplementary Information was inadvertently published previously. This has now been corrected; the PDF version of the paper was correct from the time of publication.

