# Supplementary material for: Characterization of slow cycling corneal limbal epithelial cells identifies putative stem cell markers
Source: Sci Rep. 2017 Jun 19;7:3793. doi: 10.1038/s41598-017-04006-y (PMC5476663; doi:10.1038/s41598-017-04006-y)
Supplement: Supplementary file 1 — Characterization of slow cycling corneal limbal epithelial cells identifies putative stem cell markers. [file 41598_2017_4006_MOESM1_ESM.doc]

**Characterization of slow cycling corneal limbal epithelial cells identifies putative stem cell markers.**

**Sartaj, R. _1_, Zhang, C. _2_, Wan, P., Pasha, Z. _1_, Guaiquil, V. _1_, Liu A. _2_, Liu, J. _2_, Luo_1_, Y. Fuchs, E_3_ and Rosenblatt, M_1_.**

**Corresponding Author:**

Mark I. Rosenblatt, MD, PhD, MBA

Professor and Head Department of Ophthalmology & Visual Sciences

Email: mrosenbl@uic.edu

**SUPPLEMENTARY FIGURE 1: K5Tta x TRE-H2BGFP exhibit ocular defects in around 50% of progeny**

(A) Slit lamp imaging of WT and transgenic mice. (A,a) WT FVB mouse strain shows a clear cornea devoid of vessels (n=12). (A,b) The parent transgenic strain K5Tta is normal and clear (n=8). (A,c) the second parent strain TRE-H2BGFP mouse cornea is normal (n=35) and devoid of vessels. (A,d) Around half of the progeny of the double transgenic K5Tta x TRE-H2BGFP is clear and normal (n=150) and the other half shows different degrees of corneal abnormalities such neovascularization, conjunctivalization and opacity, and in (A,e) depicts a cornea showing vascularization and conjunctivalization over the entire surface (n=148). (B) PAS staining of the WT cornea is normal and devoid of goblet cells (n=2). At 0 d or after 21d chase, slit lamp imaging shows clear corneas with no goblet cells on the corneal surface (n=2). After 42 d and 91 d chase, in corneas that showed no abnormalities by slit lamp imaging, there were no goblet cells on the cornea (n=3 and n=2, respectively). After 56 d chase abnormal corneas were infiltrated with vessels and conjunctiva overgrowth (as seen in Sup. Fig.1Ae) (n=4), showing goblet cells over the surface of the cornea as indicated by the black arrows. (C) Fluorescent microscopy of a K5Tta x TRE-H2BGFP clear corneal flatmount after 28 d doxycycline administration showing peripheral and limbal GFP localization (n=4). The distribution of GFP^+^ cells in an abnormal and opaque cornea with vessels after 35 d chase was located both at the periphery and the central cornea that included GFP^+^ aggregates (n=4). At 42 d chase, the distribution of GFP^+^ cells is seen both limbally and centrally, with cells migrating into the central area (n=7). At 49 d chase, the GFP^+^ cells are both limbally localized and aggregates of GFP are observed on the inferior section of the cornea (n=5). (D) Expression level of selected genes from the RNA-Seq data obtained by comparing GFP^-^ and GFP^+^ cells isolated from normal, clear corneas at 91 d chase (n=5) and abnormal corneas at 84 d chase (n=7). e, epithelium, s, stroma. Scale bar in B=100 µm; white arrows in C point to the GFP^+^ cells; scale bars in C=200μm. Bar chart color in D: Purple, abnormal GFP^-^, Yellow, abnormal GFP^+^, Black, GFP^-^, Green, GFP^+^.

**SUPPLEMENTARY FIGURE 2: Control groups for FACS at 28d, 42d and 91d chase**

FACS analysis of WT CD1 corneal epithelial cells used as a control group to pair with each experimental chase period. (A) When paired with 28 d chase, the experimental group detected 0.23% GFP^+^ cells (n=2). (B) When paired with the 42 d chase, the experimental group detected 0.12% GFP^+^ cells (n=2). (C) When paired with the 91d chase, the experimental group detected 0.66% GFP^+^ cells (n=3).

**SUPPLEMENTARY FIGURE 3: Immunofluorescence staining on WT adult cornea sections of potential stem cell candidate markers selected from RNA-Seq analysis.**

(A) Sox9 nuclear staining on WT sections of murine tissue was detected in few central basal cells and limbal basal epithelial cells. (B) Krt17 cytoskeletal staining was absent in the central cornea and present at the limbus only including the basal layer. (C) Actn1 cytoskeletal expression was localized to few basal epithelial cells in the central cornea and detected significantly in the limbal basal corneal epithelial cells. (D) Anxa3 cytoplasmic expression was in few central and limbal epithelial basal cells. (E) Frz7 transmembrane expression was not detected in the central cornea, but localized to the limbal basal cells (n=4 for all). Scale bars in A low mag = 1000 µm, high mag = 100 µm; B&E= 50 µm; C, D &F = 100 µm (same scale for panels). Red, candidate gene expression; Blue, DAPI.


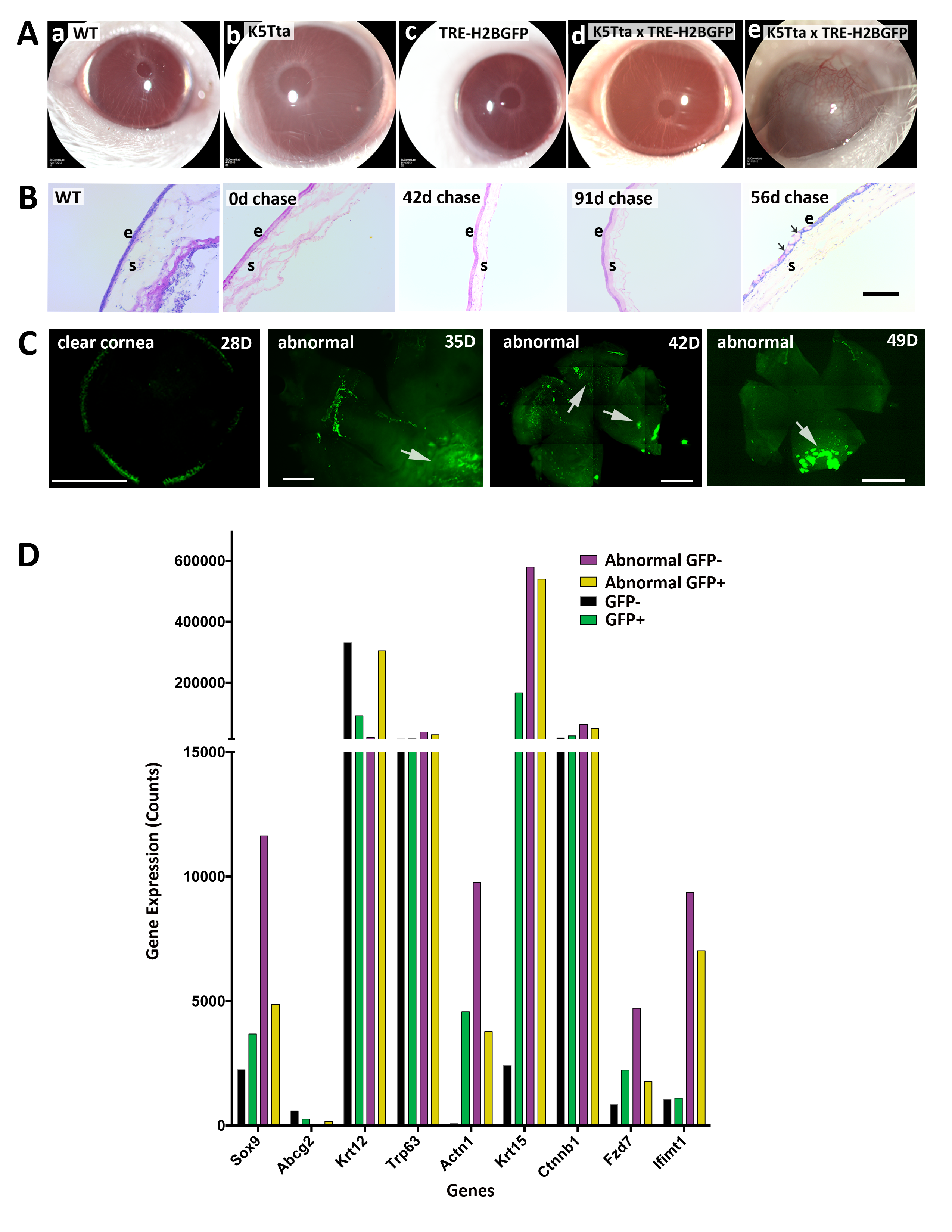


**SUPPLEMEMTARY FIGURE 1**


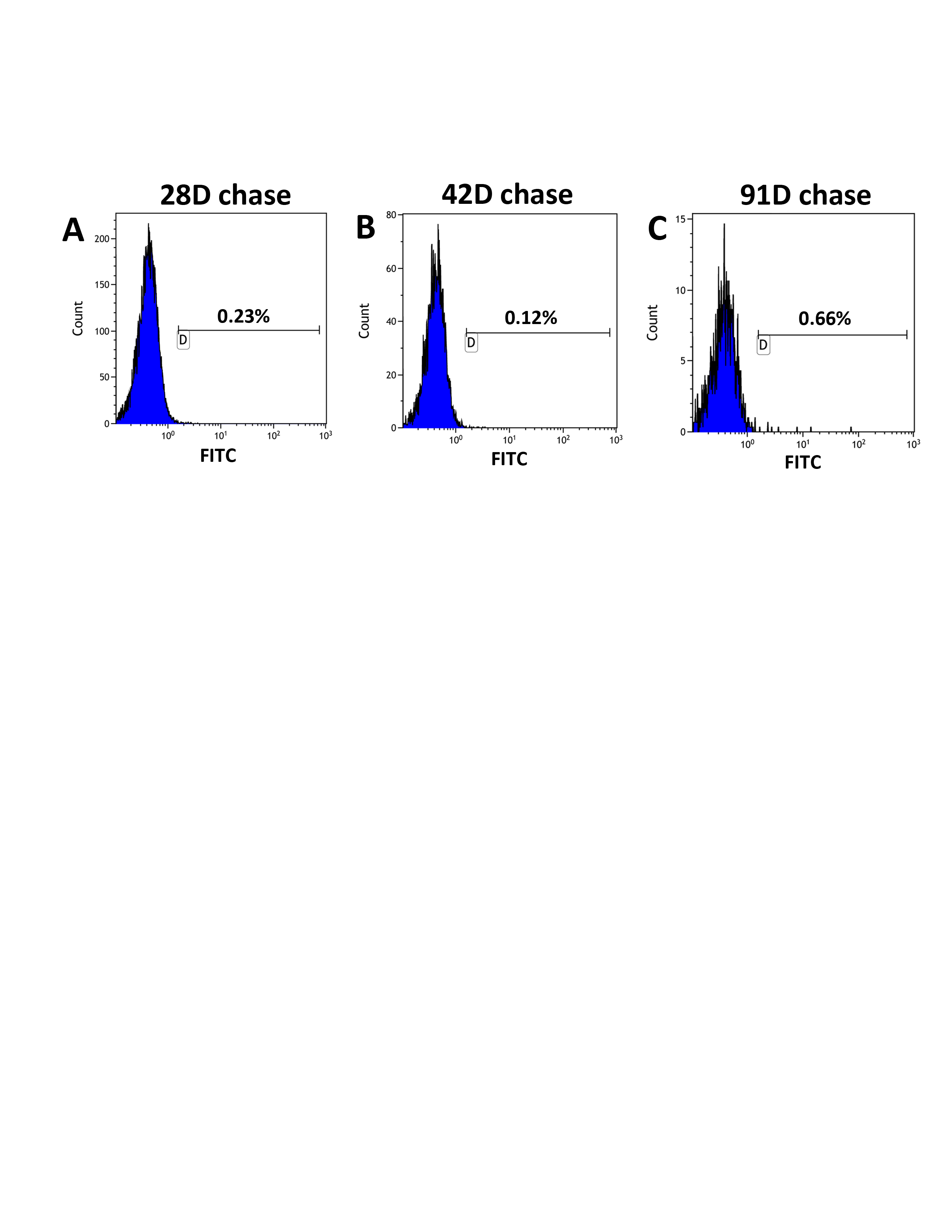


**SUPPLEMENTARY FIGURE 2**


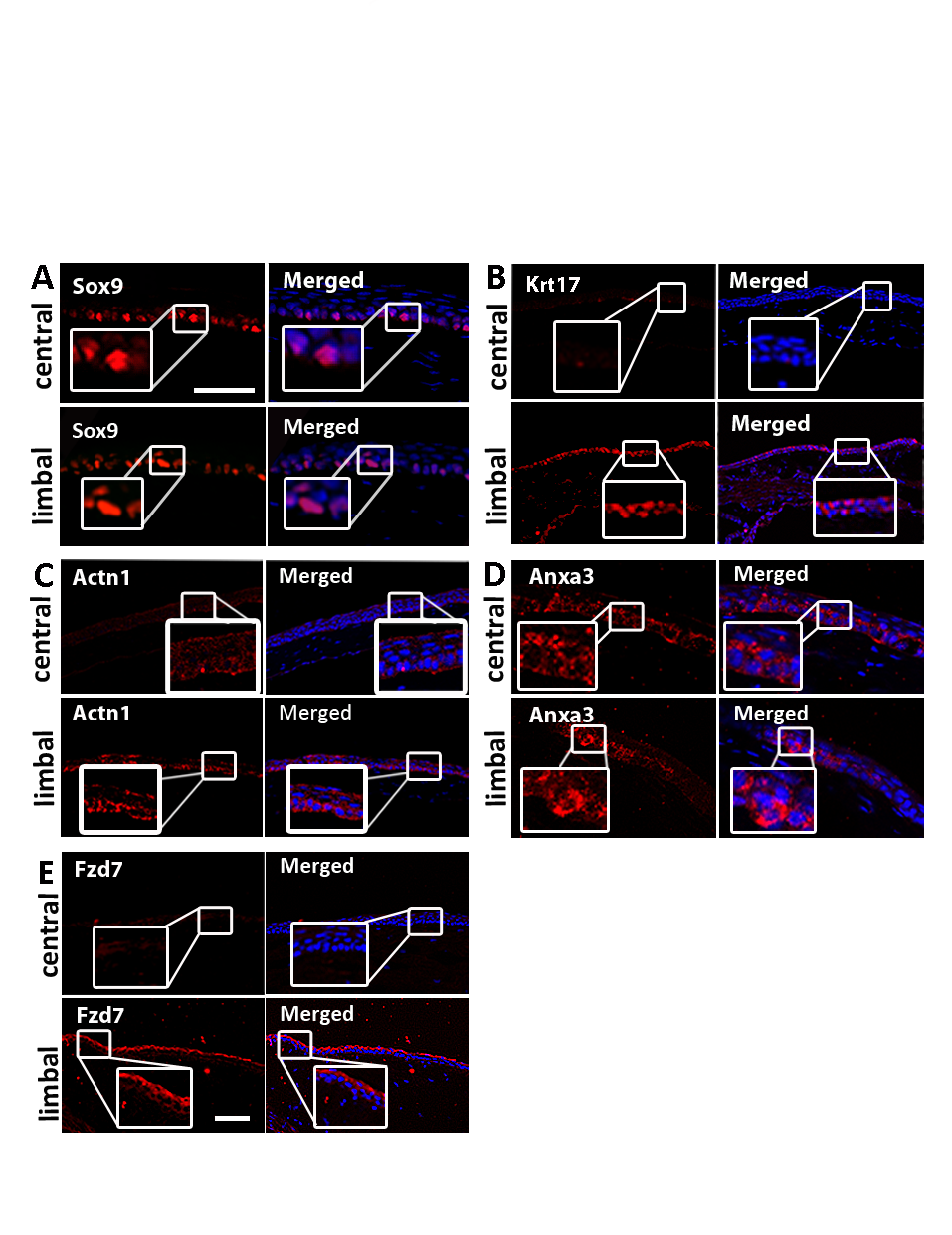


**SUPPLEMENTARY FIGURE 3**
